# Energy drink: the consumption prevalence, and awareness of its potential health implications among commercial drivers in the Ho municipality of Ghana

**DOI:** 10.1186/s12889-020-09421-x

**Published:** 2020-08-27

**Authors:** Emmanuella Yayra Saku, Peter Nuro-Ameyaw, Priscilla Cecilia Amenya, Fidelis Mawunyo Kpodo, Paul Esua- Amoafo, Nii Korley Kortei

**Affiliations:** Department of Nutrition and Dietetics, University of Health and Allied Sciences, PMB 31, Ho, Ghana

**Keywords:** Energy drink, Commercial bus drivers, Daily consumption, Reasons

## Abstract

**Background:**

Consumption of energy drinks has become an escalating global public health problem. The work schedule and irregular sleeping habits of commercial bus drivers make them highly susceptible to getting fatigued, hence most of them consume energy drinks as a fatigue management strategy. However, consumption of energy drinks produces numerous psychomotor side effects that if consumed among drivers puts the traveling public in danger of road accidents. This study sought to assess the prevalence of energy drink consumption and awareness of associated potential health problems among commercial long-distance bus drivers operating from the Ho municipality.

**Methods:**

The study population comprised about 266 commercial bus drivers. This was a cross-sectional study involving 132 participants who completed a structured questionnaire on the participants’ socio-demographic characteristics, frequency of consumption and reasons for consumption. It also included questions to assess the knowledge of the ingredients and side effects of energy drinks. Respondents were selected using a convenience sampling technique. Descriptive analysis and Chi-square test of association were used to analyse the data.

**Results:**

A majority (62.1%) of the drivers had more than 10 years of commercial driving experience. A 75% energy drink consumption prevalence was recorded with driving performance enhancement (78.8%) as the predominant reason for consumption. 7–10 bottles per week were consumed by most (32.2%) of the drivers. Also, 72.0% had poor knowledge of the side effects linked with energy drink consumption as well as the ingredients for preparation.

**Conclusion:**

Energy drinks were consumed by the majority of the drivers at the Ho main bus terminal of which most of the drivers had poor knowledge of the potential health problems linked with the consumption of these drinks. The consumption of energy drinks was observed to be higher among the drivers with lower education levels, higher monthly income and those who worked long hours in a day. The Ghana National Road Safety Commission (GNRC) in collaboration with other private road transport unions in Ghana should organize regular seminars for commercial bus drivers on the potential dangers and effects associated with energy drink consumption.

## Background

Consumption of beverages containing stimulant drugs, chiefly caffeine referred to as energy drinks have become an escalating global public health problem [1, 2]. That notwithstanding, it is a multibillion-dollar market that has experienced dynamic global growth in popularity over the years and is considered one of the fastest-growing segments of the beverage market [3, 4]. There are many brands, flavours and sizes [5] designed to give an “energy boost” to the drinker by a combination of stimulants and energy boosters [6].

Energy drinks are marketed on their supposed improvement in mental or physical performance [1, 7] and promoted within many subpopulations like commercial bus drivers, athletes and college students [8]. Many people consume energy drinks in unlimited quantities, without any regard for any adverse health implications the chemical composition of the energy drinks poses [9, 10]. Unfortunately, there are also no restrictions for sale to caffeine sensitive persons [10].

Buxton and Hagan [11], identified that in Ghana, drivers and conductors of commercial buses are high consumers of energy drinks. Commercial long-distance bus drivers are professional drivers who travel a distance of 100 km - 140 km or more regularly [12]. They are predominantly males and by nature of their work usually have irregular sleeping habits since they leave home early in the morning, return late or even do not return until the next day. As a result of their work schedule and irregular sleeping habits, they are highly susceptible to getting fatigued [13]. Many professional drivers report increased levels of sleepiness and are involved in a disproportionately high number of fatigue-related accidents, with around 40% of sleep-related accidents involving commercial bus drivers [14].

Road accident is ‘a global tragedy’ with ever-rising trend [15]. According to the World Health Organisation (WHO), every year, nearly one million people are killed, three million are severely disabled for life and thirty million are injured in road traffic accidents [16]. Across the world, the major causes of road accidents include faulty vehicles, careless/reckless driving, speeding, driver fatigue / inadequate sleep, drunk driving and other drug effects [17]. Fatigue especially is a major concern to professional drivers [18] and is ranked the 4th causing factor of road traffic accidents [19].

Driving performance is impaired when a driver is fatigued or sleepy while driving and this results in increased reaction time, reduced attention and compromised driver ability to control the vehicle [20]. For this reason, some commercial long-distance drivers consume energy drinks as a fatigue management strategy [21]. However, the consumption of energy drinks produces side effects such as severe fatigue from withdrawal, dizziness, insomnia, muscle tremors, nervousness, headache, irritability in drivers [22] which puts the traveling public in danger of road accidents.

According to Scuri and colleagues [23], the health risks associated with energy drink consumption are primarily related to their caffeine content. These health risks include reduced sleep duration and sleep quality, manifested by increased wake time after sleep onset and decreased the proportion of deep sleep [24, 25]. The subsequent consumption of energy drinks in reaction to the feeling of tiredness on the following day creates a vicious cycle of energy drink consumption and poor sleep quality resulting in recurrent fatigue [6].

Nonetheless, messages about the risks and potential undesirable effects associated with the use of energy drinks among drivers take a back seat. This being the case, commercial long-distance bus drivers may believe that energy drinks are harmless and, as a result, consume them in large quantities to get the desired effects [9], hence the need to assess the occurrence of energy drink consumption and awareness of associated potential health problems among commercial long-distance bus drivers operating from the Ho municipality.

Education on all aspects of energy drink consumption needs to become a priority, to ensure both wellness and safety especially among consumers [26, 27].

Additionally, very little attention is given to energy drink use among commercial drivers in Ghana. This research investigated energy drink use among commercial drivers in the Ho – Volta region of Ghana. Findings from the research will contribute to knowledge on public education focusing on creating awareness on road safety and health effects linked with energy drink use.

The main aim was to assess the occurrence of energy drink consumption and awareness on associated potential health problems among commercial long-distance bus drivers operating from the Ho municipality.

The study assessed the prevalence of energy drink consumption, the pattern of consumption and reasons for the consumption among commercial bus drivers in the Ho municipality. Awareness on side / adverse health effects associated with energy drink consumption among the commercial bus drivers was also assessed.

## Method

### Study area

Ho Municipal is one of the twenty-five (25) Municipalities and Districts in the Volta Region of Ghana. The Municipality is also the administrative capital and a commercial hub of the region. The municipality consists of seven hundred and seventy-two (772) communities and a land Size of 2660 sq. according to the Ghana Statistical Service.

### Study site

The Ho main transport terminal was the study site for this research. Below is the geographical location of the Ho main transport terminal on a google map.

### Study population

There are three (3) different organizations operating in the Ho main transport terminal, namely; Ghana Private Road Transport Union (GPRTU), Progressive Transport Owners Association (PROTOA), and Cooperative. PROTOA and Cooperative have about thirty-five (35) and twenty-nine (29) drivers respectively, registered under them. GPRTU has been divided into six (6) branches each of which has averagely twenty-five (25) registered drivers. There are also about fifty-two (52) non-registered drivers (floating drivers) according to the authorities of the transport unions.

The study population included commercial bus drivers in the Ho main transport terminal irrespective of the transport union they belonged to.

### Inclusion and exclusion criteria

Only commercial bus drivers in the Ho main station who are above 18 years of age and ply routes to areas outside Ho were eligible to partake in the study. Drivers who drive within Ho were excluded.

### Study design

The study design was cross-sectional. This helped describe or determine the prevailing characteristics of interest at a specific point in time.

### Sample size determination

A sample size (n) of 132 participants was determined by inputting a population size (N) of 266 commercial bus drivers, an expected frequency of 78% [28], a confidence limit of 5% and a confidence level of 95% into the EpiInfo sample size calculator (android version). This calculator works based on the Modified Cochran Formula for sample size calculation in smaller populations [29].

${n}_o=\frac{{\mathrm{Z}}^2\mathrm{pq}}{{\mathrm{e}}^2}$ where n˳ = Estimated sample size, Z = z value (1.96 for 95% confidence level), e = confidence interval (0.05), *p =* expected frequency (0.78), q = 1-p

$$ n=\frac{n_{\mathrm{o}}}{1+\frac{\left({n}_o-1\right)}{N}} $$

### Sampling technique

A convenience sampling technique was used to recruit eligible respondents in the study. The time spent by the drivers at the bus terminal is inconsistent since their daily work routine involves coming to the terminal and leaving at any time of the day depending on the availability of passengers. This means, a driver could be accessible at the bus terminal only when he is waiting to transport passengers. In light of this, the drivers were sampled based on easy accessibility and availability any given time when data was collected.

### Data collection techniques

A structured questionnaire was used to obtain information from the respondents. The main media of communication in administering the questionnaire were English, Ewe, and Akan. Nevertheless, help from an interpreter was sought in the case of a language barrier. The questionnaire comprised three (3) different sections. Section A focused on participants’ socio-demographic information, section B contained multiple-choice questions on whether or not participants consume energy drinks, their frequency of consumption as well as their reasons for consumption. Section C also comprised several questions about respondents’ general knowledge on energy drinks which included composition and adverse health effects of energy drinks.

### Data analysis

Data collected was cleaned to detect and remove inaccurate and incomplete data. Completeness and consistency of the data were checked and entered into Statistical Package for Social Sciences (SPSS) data analysis software version 20 for analysis. Descriptive statistics obtained through descriptive analysis helped to ascertain the prevalence of energy drink consumption as well as the reasons, pattern, and frequency of consumption of energy drinks. Chi-square analysis (Likelihood ratio Chi-square test) was used to assess the relationship between energy drink consumption and the awareness of commercial bus drivers on the side / adverse effects linked with the use of these drinks.

Results were presented in the form of tables, graphs and pie charts to facilitate interpretation. Also, threshold of significance was set at < 0.05 (*p* < 0.05).

## Results

The data gathered from the respondents were analysed under the following headings; socio-demographic characteristics, energy drink consumption or use, reasons for consumption, a pattern of consumption and general knowledge on energy drinks. Frequency and percentage were calculated for each categorical variable. *P*-value was used to determine statistically significant associations.

### Socio-demographic characteristics of the commercial bus drivers

The socio-demographic characteristics of the study participants are presented in Table 1. One hundred and thirty-two (132) respondents were included in the study. All the respondents were males with the dominating age range being between the ages of 36–45 (34.1%) years. Also, most of them (80.3%) were Ewes and Christianity was the predominant religion (88.6%) of the drivers. Findings also revealed that more than half of the respondents (68.9%) were married. More than half (58.3%) of them had an educational level of SHS / Vocational training / Technical training whereas 6.8% had no formal education. Concerning years of commercial driving experience, the majority (62.1%) had more than 10 years of experience and 47.2% worked for between 4 and 6 h in a day.

**Table 1:** Socio-Demographic Characteristics of Commercial Bus Drivers

Parameter	Frequency	Percent
Total	132	100.0
**Age**		
18–25	12	9.1
26–35	38	28.8
36–45	45	34.1
> 45	37	28.0
**Gender**		
Male	132	100.0
**Ethnicity**		
Ewe	106	80.3
Akan	14	10.6
Northerner	12	9.1
**Religion**		
Christian	117	88.6
Muslim	15	11.4
**Marital status**		
Single	35	26.5
Married	91	68.9
Widowed	6	4.5
**Educational Level**		
JHS	24	18.2
SHS / Voc / Tech	77	58.3
Tertiary	22	16.7
None	9	6.8
**Income / month**		
< ghȼ100	18	13.6
ghȼ100–500	62	47.0
ghȼ600–1000	29	22.0
> ghȼ1000	23	17.4
**Commercial Driving Experience (years)**		
< 1	4	3.0
1–3	8	6.1
4–6	19	14.4
7–10	19	14.4
> 10	82	62.1
**Working Hours/Day**		
< 3	17	12.9
4–6	62	47.0
7–10	29	22.0
> 10	24	18.2

Data presented as frequency and corresponding percentage

### Prevalence of energy drink consumption

**Fig. 1:**
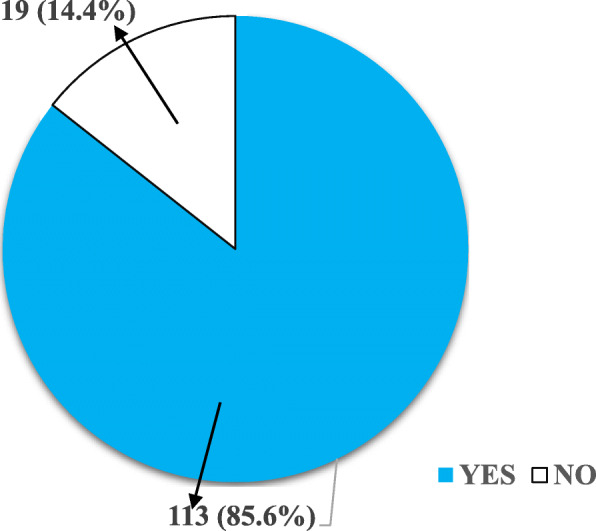
Number of Participants Who Have Ever Consumed Energy Drinks. Data presented as frequency and (corresponding percentage)

**Fig. 2:**
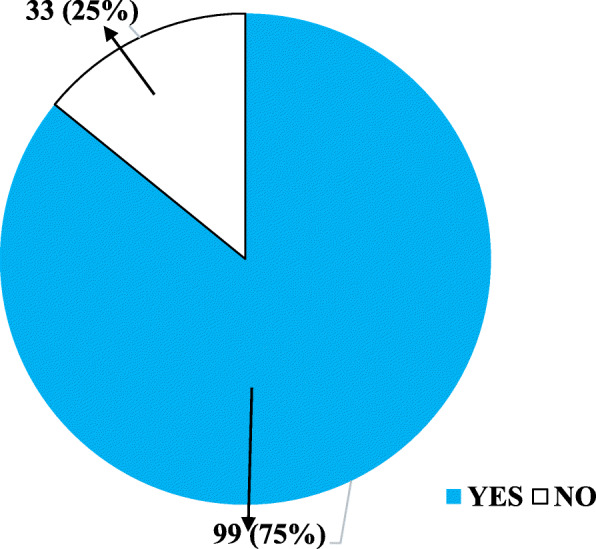
Number of Respondents Who Presently Consume Energy Drinks. Data presented in frequency and (corresponding percentage)

The results of the prevalence of energy drink consumption among the participants were in two categories. Those that have ever consumed energy drink before as well as those that are currently consuming the drink as demonstrated in Figs. 1 and 2 respectively.

### Number of drivers who currently consumed energy drinks

Most of the participants 113 (85.6%) had ever consumed energy drinks as shown in Fig. 1.

Out of the 113 drivers who had consumed energy drinks before, 99 currently consume the drinks. The number who currently took energy drinks expressed as a percentage of the total number of respondents surveyed (132) gave the prevalence of consumption. This implies that the prevalence of energy drink consumption among commercial bus drivers in the Ho main transport terminal is 75%. Figure 2 illustrates the prevalence of energy drink consumption.

### Reasons for consuming energy drinks

**Fig. 3:**
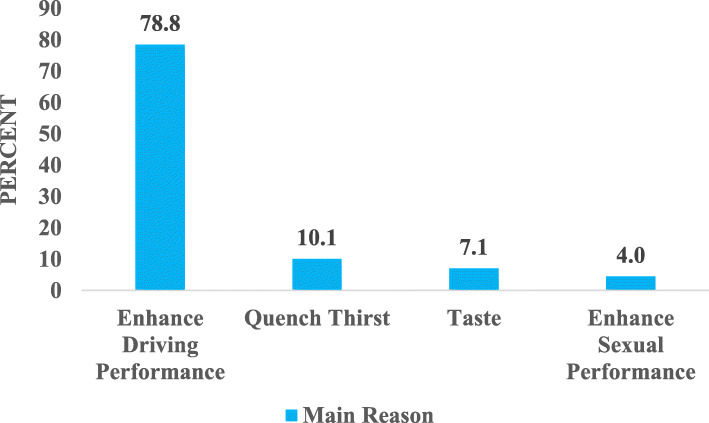
Reasons and Motivations for Consuming Energy Drinks

The study results showed that there is a myriad of reasons or motivations for consuming energy drinks among drivers. The main reasons for which respondents were currently consuming energy drinks are seen in Fig. 3. It was found that most of the drivers (78.8%) consumed energy drinks to enhance driving performance. Thus, to stay awake while driving (41.4%), to reduce fatigue (17.2%), for an energy boost (17.2%) as well as for mental enhancement (3.0%) as seen in Fig. 3. Also, to quench thirst (10.1%), its pleasant taste (7.1%) and for sexual enhancement (4.0%) were some of the other reasons for which the drivers consumed energy drinks.

**Fig. 4:**
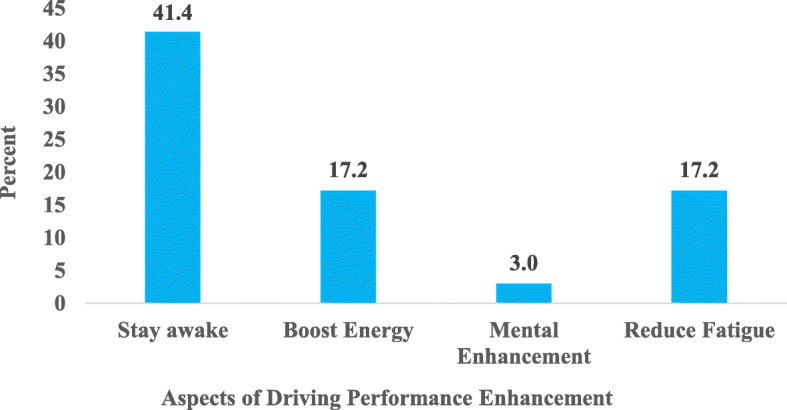
Factors Affecting Driving Performance

Figure 4 illustrates the aspects of driving performance the drivers sought to enhance. Intending to enhance driving performance, the drivers consumed energy drinks to stay awake, boost energy, enhance mental performance, and reduce fatigue while driving.

### A pattern of energy drink consumption

#### Initial introduction of drivers to energy drinks

**Fig. 5:**
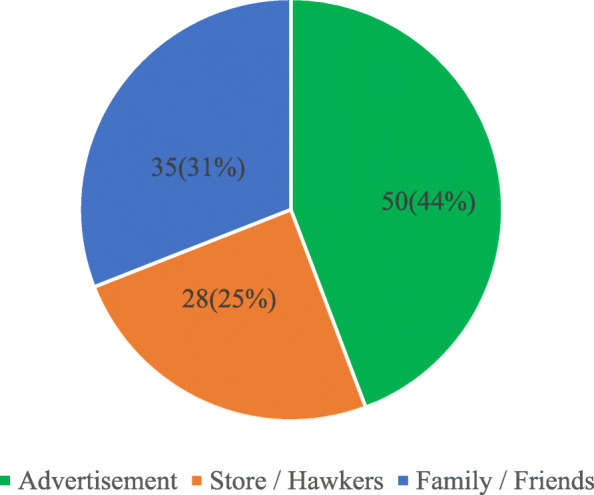
Mode of Introduction to Energy Drinks

An investigation to ascertain the prevailing mode of introduction to energy drinks revealed that advertisement was the highest mode of drivers’ introduction. While 50 (44.2%) were first introduced through advertisement, 35 (31.0%) and 28 (24.8%) were introduced through sales at stores or by family and friends respectively (Fig. 5).

#### Time of day energy drink is consumed among drivers

**Fig. 6:**
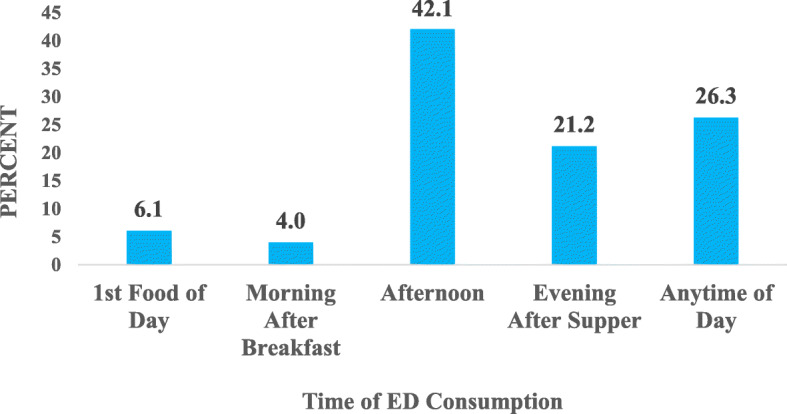
Time of Day of Energy Drink Consumption among Drivers. ED = Energy drink

Over four out of ten (42.4%) consumed energy drinks in the afternoon, followed by 26.3% consuming at any time of the day. Aside from that, the drinks were also consumed in the morning either as the first food of the day (6.1%) or after breakfast (4.0%). Figure 6 shows the common times in the day during which energy drinks are taken among drivers.

#### Frequency of energy drink consumption among drivers

**Fig. 7:**
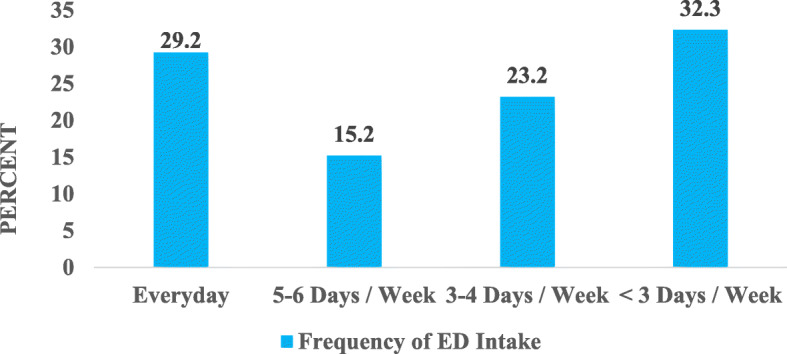
Frequency of Energy Drink Consumption among Drivers. ED = Energy drink

Almost 30% of the drivers were daily consumers of energy drinks at the time of the study. Among the remaining, 32 (32.3%) consumed the drinks less than 3 days in a week, 15 (15.2%) for 5–6 days in a week and 23 (23.2%) for 3–4 days in a week. Figure 7 displays the frequency of energy drinks consumption among drivers.

#### Number of bottles / cans of energy drinks consumed in a week by drivers

**Fig. 8:**
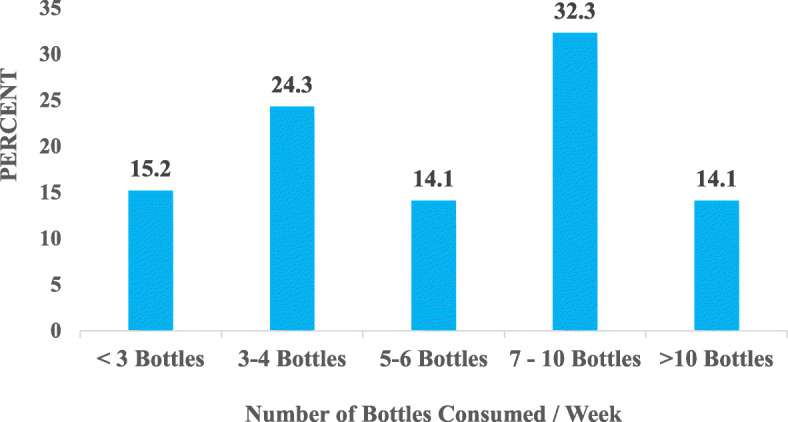
Number of Bottles / Cans Consumed Per Week

Figure 8 shows that the majority of the drivers (32.3%) consumed 7–10 bottles of energy drinks in a week. Also, 14.1, 24.3, and 15.2% consumed 5–6, 3–4 and less than 3 bottles respectively in a week. 14.1% also consumed more than 10 bottles in a week.

Energy drink consumption was differentiated across the various age groups, educational levels and years of commercial driving experience. Results showed that, more than 80% of drivers of each age group consumed energy drinks. Across the various age groups, there was no significant difference in the likelihood to consume energy drinks. As the educational level declined, the number of drivers that consume energy drinks also reduced. There was also no significant difference between the numbers of energy drink consumers across the various years of commercial driving experience.

#### Working hours per day and time of day energy drink is consumed

Findings from the study showed that drivers who work for less than 3 h were more likely to drink energy drinks as their first food of the day whereas those who work more than 10 h a day were more likely to consume the drink any time of the day. Most of those who work for 4–6 and 7–10 h and consumed energy drinks in the afternoon (Table 2).

**Table 2:** Working Hours Per Day and Time of Day Energy Drink Is Consumed

Parameter	Working Hours / Day				
Time of ED Consumption	< 3	4–6	7–10	> 10	Total
**1st Food of Day**	4 (40.0)	0	0	2 (7.4)	6 (6.1)
**Morning After Breakfast**	0	4 (8.7)	0	0	4 (4.0)
**Afternoon**	4 (40.0)	20 (43.50)	10 (63.5)	8 (29.6)	42 (42.4)
**Evening After Supper**	2 (20.0)	8 (17.40)	4 (25.0)	7 (25.9)	21 (21.0)
**Any time of Day**	0	14 (30.4)	2 (12.5)	10 (37.0)	26 (26.3)
**Total**	10 (100)	46 (100)	16 (100)	27 (100)	99 (100)

Data presented as frequency and corresponding percentage

*ED* Energy drink

#### Income and number of bottles / cans of energy drinks consumed

Table 3 shows that the number of bottles/cans of energy drinks a driver consumes per week was more likely to increase as the income increased. Drivers who earn less than Ghȼ 100 were more likely to consume 5–6 bottles in a week whereas Ghȼ 100–500, Ghȼ 600–1000 and > Ghȼ 1000 earners were more likely to consume 7–10 bottles in a week.

**Table 3:** Drivers’ Income and Number of Bottles / Cans of Energy Drinks Consumed

Parameter	Income				
Bottles / Week	<Ghȼ100	Ghȼ 100–500	Ghȼ 600–1000	>Ghȼ 1000	Total
**< 3 Bottles / Week**	0	11 (21.2)	1 (5.6)	3 (18.8)	15 (15.2)
**3–4 Bottles / Week**	4 (30.8)	13 (25.0)	5 (27.8)	2 (12.5)	24 (24.2)
**5–6 Bottles / Week**	5 (38.5)	7 (13.5)	1 (5.6)	1 (6.2)	14 (14.1)
**7–10 Bottles / Week**	2 (15.4)	14 (26.9)	11 (61.1)	5 (31.2)	32 (32.3)
**> 10 Bottles / Week**	2 (15.4)	7 (13.5)	0	5 (31.2)	14 (14.1)
**Total**	13 (100)	52 (100)	18 (100)	16 (100)	99 (100)

Data presented as frequency and corresponding percentage

*ED* Energy drink, *ghȼ* Ghana cedis

#### Response to whether intended benefits are derived

**Fig. 9:**
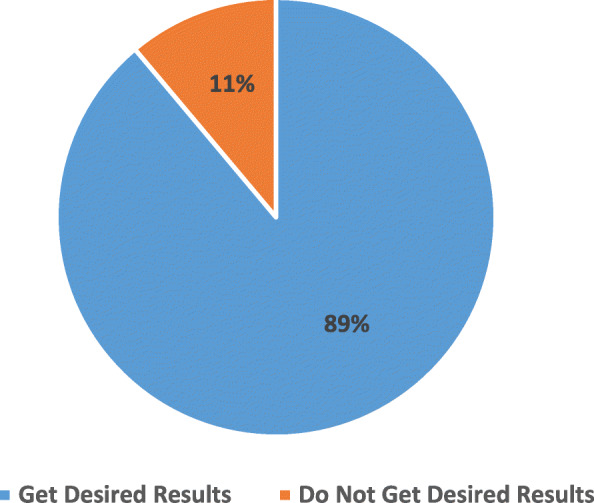
Respondents’ Responses to Getting Desired Results When They Consume Energy Drinks

Almost nine out of ten (88.9%) of the respondents who consumed energy drinks responded in affirmative when asked if they got the intended benefits or desired results after consuming the energy drinks. However, 11.1% reported not attaining their intended results. Seen in Fig. 9 are the responses to the satisfaction question.

#### General knowledge on energy drinks

**Fig. 10:**
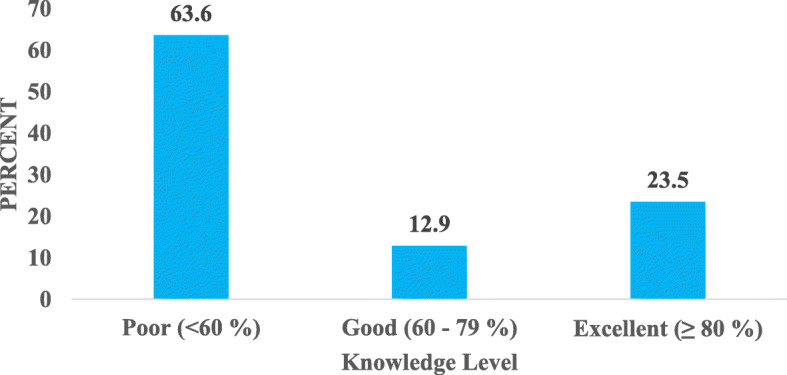
Level of Knowledge of Drivers on Energy Drinks

The level of knowledge of the drivers on the ingredients as well as potential side / adverse health effects of these drinks is illustrated in Fig. 10 Over 60% of the respondents had poor general knowledge level, 12.9% had a good general knowledge level and 23.5% had an excellent general knowledge level.

**Fig. 11:**
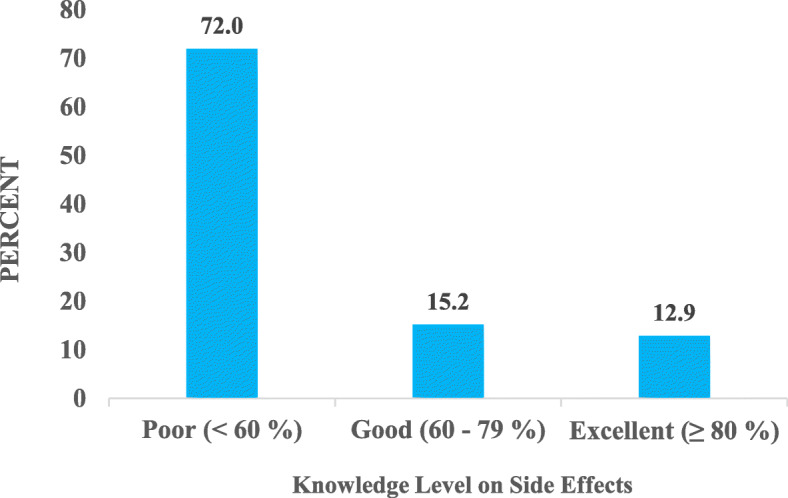
Respondents’ Knowledge on Side Effects of Energy Drink

Over 7 out of 10 respondents had poor knowledge and 12.9% had excellent knowledge on the side effects linked with energy drink intake (Fig. 11).

#### Relationship between knowledge on side effects and consumption of energy drinks

Table 4 indicates that there is no statistically significant association between drivers’ level of knowledge on side effects linked with energy drink intake and the consumption of these drinks. More than 80% of the drivers that fell under each of the knowledge categories consumed energy drinks. Irrespective of the difference in knowledge levels, there was no difference in the likelihood to consume energy drinks.

**Table 4:** Pattern of Energy Drink Consumption

ED Consumption	Age				
	**18–25**	**26–35**	**36–45**		**> 45**
Yes	9 (100)	30 (93.8)	32 (80.0)		28 (87.5)
No	0	2 (6.20)	8 (20.0)		4 (12.5)
Total	9 (100)	32 (100)	40 (100)		32 (100)
	**Educational Level**				
	**SHS / Voc / Tech**	**Tertiary**	**JHS**		**None**
Yes	57 (90.5)	9 (52.9)	24 (100)		9 (100)
No	6 (9.5)	8 (47.1)	0		0
Total	63 (100)	17 (100)	24 (100)		9 (100)
	**Commercial Driving Experience (years)**				
	**< 1**	**1–3**	**4–6**	**7–10**	**> 10**
Yes	2 (100)	7 (100)	11 (73.3)	17 (100)	62 (86.1)
No	0	0	4 (26.7)	0	10 (13.9)
Total	2 (100)	7 (100)	15 (100)	17 (100)	72 (100)
Association Between Knowledge on Side Effects and Energy Drink Consumption					
	**Knowledge of Side Effects**				
	**Poor (< 60%)**	**Good** **(60–79%)**	**Excellent (≥80%)**	**Total**	***p*-value (likelihood ratio)**
Yes	68 (87.2)	16 (88.9)	15 (88.2)	99 (87.60)	0.977
No	10 (12.8)	2 (11.1)	2 (11.8)	14 (12.4)	
Total	78 (100)	18 (100)	17 (100)	113 (100)	

Data presented as frequency and corresponding percentage

*ED* Energy drink

## Discussion

This study was aimed at assessing the occurrence of energy drink consumption among commercial long-distance bus drivers operating from the Ho municipality and also investigating their awareness of potential health problems associated with the consumption of these energy drinks. Information on the consumption status of the drivers, the reasons for consumption, the pattern of consumption and general knowledge on energy drinks was obtained from 132 participants using a structured questionnaire.

### Socio-demographic characteristics of the commercial bus drivers

Aside all the participants being males, 36–45 (34.1%) years was the leading age range. According to Achulo et al. [30, 31], most commercial drivers in Ghana have been reported to be within the age range of 30–50 years which could also explain the majority of the drivers being between 36 and 45 years of age. The least age range (9.1%) being within 18–25 years suggest that many vehicle owners consider long years of experience before they hand over their vehicles to a driver for commercial purposes. The absence of ages less than 18 years implies that the Drivers and Vehicle Licensing Authority (DVLA), a regulatory body in Ghana, sticks to the law governing the issuance of driver’s license to people above 18 years only [32]. SHS, vocational training or technical training being the dominant educational level of majority (58.3%) of the drivers suggests that, most people opt for commercial driving as an occupation as a result of their inability to further their studies to the university to get them jobs of their interest. It may also be due to limited vacancy for blue- and white-collar jobs in Ghana especially for people without very high levels of education [30]. Additionally, it was observed that 80.3% were Ewes which may be because the study was carried out in the Volta region which is inhabited mainly by Ewes. It also suggests that the routes commercial drivers use in Ghana might be influenced by ethnicity. Almost half of the drivers had a monthly income range of Ghȼ 100–500 ($17.83–89.93). This suggests that many commercial drivers may be earning around the daily minimum wage of Ghȼ 10.65 ($1.90), translated into Ghȼ 319.5 ($56.95) [33]. The low income the drivers collect may be the reason why some of them consume energy drinks, so as to boost their energy levels at the least cost. Concerning years of commercial driving experience and the number of working hours per day, most (62.1%) of them had greater than 10 years of experience and almost half of the drivers (47.0%) worked for between 4 and 6 h per day.

### Prevalence of energy drink consumption among the commercial bus drivers

This present study reveals a 75% prevalence of energy drink consumption among commercial bus drivers in Ho. Sharwood et al. [34], stipulates that commercial bus long-distance drivers have developed many strategies to improve their performance while driving, among which is the use of energy drinks. This supposed positive effect of energy drinks could explain their widespread use amongst the study participants [35]. Klu et al. [28] however observed a slightly higher prevalence of energy drink consumption of 78% among commercial bus drivers and hawkers in the Tema Municipality of the Greater-Accra region, Ghana. In line with this, Buxton and Hagan [11] reported that commercial bus drivers are among the high consumers of energy drinks in Ghana.

On the other hand, findings by Sharwood et al. [21] in a case-control study to investigate the existence of an association between caffeinated beverage use and the risk of crash in long-distance commercial bus drivers, reported a lower prevalence of energy drink consumption, thus, 14 and 6% among the controls and cases respectively.

The difference in prevalence among the various studies could be attributed to the variations in population and sample sizes as well as the types of study.

### Reasons for consuming energy drinks among the commercial bus drivers

Respondents of this present study gave varied reasons for consuming energy drinks which included enhancing driving performance (keeping awake while driving, for an energy boost, reduce fatigue and mental enhancement), to quench thirst, for the taste, as well as sexual enhancement. The most predominant reason given by almost 8 out of 10 drivers (78.8%) was to enhance driving performance. This is in line with findings by Acevedo and colleagues [36], in which the majority (61%) of the study participants took energy drinks for enhanced performance. This suggests that most drivers overwork and need to be educated to take rests to reduce fatigue-related accidents on the roads. According to Sharwood et al. [34], long-distance drivers of commercial vehicles routinely experience monotonous and extended driving periods in a sedentary position. This, often combined with the disruption to circadian rhythms linked to the common requirement of night driving, has been associated with wake time drowsiness and increased fatigue. This could also account for most of the driver’s energy drinks consumption to increase energy and counter sleepiness and fatigue, thereby enhancing driving performance.

The percentage of drivers who consumed energy drinks due to the taste in this present study was 7.1%. Similarly, the pleasant taste was a reason given by participants of a study conducted by Klu et al. [28]. According to Giles and colleagues [37], most energy drinks contain sweeteners like glucose and other flavorings which contribute to improving their taste.

Energy drinks are consumed to quench thirst or as sexual enhancer and implied that little or no attention is given to the quantity or even frequency of consumption. This can be attributed to the unchecked and inadequate regulatory control of caffeinated energy drinks in Ghana [28].

### The pattern of energy drink consumption among the commercial bus drivers

Results of this study showed that participants were first introduced to energy drinks either through advertisement, recommendations from family or friends, exposure by hawkers or at convenience stores. The predominant mode of introduction was advertisement (44.2%). This was followed by recommendations from family/friends which constituted 31.0%. Initiating energy drink use through the influence of advertisements is not surprising since the adverts on energy drinks are very appealing, having individuals with fast-paced lifestyles and looking for an energy boost as their target. These could include drivers wanting to sustain long hours of driving [38]. Manufacturers of these drinks also advertise their products by sponsoring extreme sports like race car driving [27] and this manner of advertisement can easily appeal to commercial bus drivers who seek heightened driving performance when behind the steering wheel.

Reid et al. [38] revealed from their study that 31.2% of the study participants were introduced to energy drinks through recommendations from friends and family, similar to 31.0% this study found, followed by 30.6% introduced through advertisement. This suggests that close relatives and associates play a critical role in the eating pattern of these drivers.

Taking into consideration the quantity and frequency of energy drink intake, this present study identified that almost a third of the participants consumed 7 to 10 bottles of energy drinks per week with less than 3 bottles being the least number of bottles consumed per week. This was much lower than the 7 to 21 bottles, 28 to 42 bottles and 49 bottles of energy drinks per week consumed by 94, 2 and 4% of participants respectively reported by Klu et al. [28]. The reasons for this sharp disparity could be attributed to brisk economic activities in Tema compared to Ho. Tema has the major export and import harbour in Ghana. Additionally, it is in a close proximity to Accra, Ghana’s capital city. The economic activities in Ho is predominantly petty trading and subsistence farming. This suggests that the busier the place the drivers operated, the longer the hours the drivers sat behind the wheels and this influenced the quantities of energy drink intake. The intake of a high number of bottles of energy drinks per week could be due to the addictive nature of caffeine and other stimulants such as taurine, guarana ...etc. in these drinks [39]. Moreover, the results of this study showed that the number of bottles of energy drinks the drivers consume is related to the income they earn. Drivers who earned less than Ghȼ 100 ($17.83) were more likely to consume 5–6 bottles in a week whereas Ghȼ 100–500 ($17.83–89.93), Ghȼ 600–1000 ($106.95–178.25) and > Ghȼ 1000 ($178.25) earners were more likely to consume 7–10 bottles in a week.

This study also revealed that 29.2% of the drivers took energy drinks every day and most commonly in the afternoon. This pattern of energy drink consumption could be attributed to the quest for a cool sensation to alleviate perceived stress which is due to increased levels of heat especially from the sun, experienced during the afternoon. The perception of stress can cause reduced alertness and productivity among drivers [40]. Drivers who work for less than 3 h had a higher likelihood of consuming energy drinks as their first food of the day. This could be because they presumed the drinks would supply them with energy to proceed with the day’s activities. Those who worked more than 10 h a day were more likely to consume the drink any time of the day, probably because they might have felt fatigued at any point in time as they worked and hence resorted to energy drinks to replenish their energy. Most of those who work for 4–6 and 7–10 h consumed energy drinks in the afternoon. This suggests their work hours spanned through the afternoon or even beyond and as a result, end up consuming energy drinks with the intention of cooling the body as a result of heat from the sun.

Majority (88.9%) of the respondents also affirmed getting the desired results when they take energy drinks. This is much higher than the 57.1% reported by Klu et al. [28]. This suggests that when the drivers keep consuming higher quantities of energy drinks, they develop an addiction for the caffeine and hence require much higher quantities to obtain their desired results.

### General knowledge on energy drinks among the commercial bus drivers

Over 6 out of 10 drivers (63.6%) had relatively poor knowledge on energy drinks including knowledge on the ingredients and associated side / adverse health effects. Subaiea et al. [1] also recorded poor knowledge in energy drinks adverse effects among Saudi Arabian populace. According to Gunja and Brown [22], generally, there is a poor knowledge level on energy drink ingredients, effects and toxicity. This can be attributed to the fact that some manufactures do not have many of the ingredients and their quantities likewise warning labels on their products [5]. Also, messages about the risks and potential undesirable effects associated with energy drink use are seldomly mentioned or talked about compared with the high level of exposure of these drinks [9].

The study results also showed that there was no statistically significant relationship between consumption of energy drinks by drivers and their knowledge on the potential side / adverse health effects linked with energy drink intake. This suggests that having knowledge on the potential health problems associated with energy drink intake does not influence a drivers’ decision to consume energy drinks or not.

Our inference using the Health Belief Model suggests that the commercial bus drivers are most likely to take preventative actions if they perceive the threat of a health risk to be serious, if they feel they are personally susceptible and if there are fewer benefits than costs to engaging in it. Also, behavior change mediations will be more effective if they address these commercial bus driver’s specific opinions about vulnerability, benefits, barriers, and self-efficacy.

## Conclusion

Generally, this research revealed a relatively high prevalence of energy drink consumption among commercial bus drivers in the Ho main transport terminal. The consumption of energy drinks was observed to be higher among the drivers with lower educational levels, higher income earners as well as those who worked longer hours in a day. Most of the drivers take 7–10 bottles of energy drinks in less than 3 days per week which suggests an intake of 2 or more bottles in a day. Most of the drivers however had poor knowledge on the potential health problems linked with taking these drinks and this could contribute to consuming these drinks without caution or fear of consequent potential health problems. This in the long run may increase their risk for potential side / adverse health effects linked with energy drink consumption.

## Data Availability

The datasets used and/or analyzed during the current study are available from the corresponding author on reasonable request.

## Abbreviations

GPRTU: Ghana Private Road Transport Union
PROTOA: Progressive Transport Owners Association
WHO: World Health Organization
SHS: Senior High School
SPSS: Statistical Package for Social Sciences
UHAS: University of Health and Allied Sciences
Ghȼ: Ghana cedis
DVLA: Driver and Vehicle Licensing Authority
UHAS-REC: University of Health and Allied Sciences Review and Ethics Committee
GNRC: Ghana National Road Safety Commission
